# Evaluation of Tyrosinase Inhibitory, Antioxidant, Antimicrobial, and Antiaging Activities of* Magnolia officinalis* Extracts after* Aspergillus niger* Fermentation

**DOI:** 10.1155/2018/5201786

**Published:** 2018-11-15

**Authors:** Lichun Wu, Chihyu Chen, Chiuyu Cheng, Hang Dai, Yazhao Ai, Chiahui Lin, Yingchien Chung

**Affiliations:** ^1^Department of Logistics Engineering, Dongguan Polytechnic, Dongguan City 523808, Guangdong Province, China; ^2^Department of Tourism and Leisure, Hsing Wu University, New Taipei City 22452, Taiwan; ^3^Department of Biological Science and Technology, China University of Science and Technology, Taipei City 11581, Taiwan

## Abstract

This study intended to improve physiological characteristics of* Magnolia officinalis *bark (MOB) extracts by* Aspergillus niger *fermentation.* M. officinalis* bark was extracted using distilled water, 95% ethanol, and methanol, and it was then fermented by* A. niger*. The physiological characteristics of the fermented extracts, namely, tyrosinase inhibitory activity, antioxidant activity, antibacterial activity, and anti-skin-aging activity, were evaluated and compared with those of unfermented extracts. To determine the safety of the fermented extracts, their cytotoxicity was analyzed by measuring the cell viability of CCD-966SK and human epidermal melanocytes (HEMn) after exposure. The fermented methanol extract exhibited the highest antityrosinase activity, total phenolic content, and antioxidant activity. The total phenolic content of the extracts fermented by* A. niger* was 3.52 times greater than that of the unfermented extracts. The optimal IC_50_ values for tyrosinase inhibition and 2,2-diphenyl-1-picrylhydrazyl (DPPH) removal by the* A. niger-*fermented extracts were 30 and 12 *μ*g/mL, respectively. The fermented methanol extracts inhibited skin-aging-related enzymes such as collagenase, elastase, MMP-1, and MMP-2. Compared with the unfermented extracts, the fermented extracts also contained greater antibacterial activity against tested stains including* MRSA*. These results could be attributed to an increase in the concentration of original active compounds and the biosynthesis of new compounds during fermentation. In cytotoxicity assays, the* A. niger-*fermented extracts were nontoxic to CCD-966SK cells, even at 500 *μ*g/mL. Hence, in general, methanol-extracted* M. officinalis* fermented by* A. niger* for 72 h has the most active antioxidant, skincare, or antiaging compounds for healthy food or cosmetics applications.

## 1. Introduction

Melanin is the black pigment in hair and skin and is essential for protecting human skin against radiation. Accumulation in the epidermal layer leads to melanogenesis or skin pigmentation, and this can be undesirable [1]. Pharmacologically, melanogenesis can be controlled by inhibiting the activity of tyrosinase or other related melanogenic enzymes. Among melanogenic enzymes, tyrosinase is the rate-limiting enzyme for controlling the production of melanin [2]. The use of tyrosinase inhibitors is the most promising method for melanogenesis inhibition. Tyrosinase inhibitors specifically interact with melanogenic cells and do not lead to side effects compared with other melanogenesis inhibitors [3].

Nontoxic natural products used in formulating cosmetics and pharmaceuticals are of considerable interest. Natural products made from plant sources have been used in cosmetic applications as whitening agents and as a nutritional source [4]. Of particular interest are antioxidants in herbal extracts that possess multiple beneficial functions such as (1) preventing free radical formation and decreasing ultraviolet- (UV-) radiation-mediated oxidative damage by inhibiting the initiation or propagation of oxidizing chain reactions; (2) inhibiting tyrosinase activity or the expression of melanogenic enzymes by chelating metals at their active sites, thereby further decreasing melanin production [5, 6].

Skin aging is a complicated biochemical process; collagen and elastin degradation occur in the epidermal and dermal layers and are related to extracellular matrix (ECM) degradation. The enzymes involved in ECM degradation are matrix metalloproteinases (MMPs) such as interstitial collagenase (MMP-1) and 72-kDa gelatinase (MMP-2). Skin loses its tensile strength due to ECM degradation; thus, MMPs are considered to be involved in wrinkle formation [4]. Moreover, extrinsic factors such as exposure to UV radiation lead to the activation of collagenase, elastase, and tyrosinase, thus resulting in skin aging, wrinkle formation and melanin production [7, 8]. Therefore, exploring fermented herb extracts that have beneficial effects to prevent skin aging is important.

Fermentation may increase the physiological and biochemical activities of biological substrates by modifying their naturally occurring molecules [3]. Moreover, fermentation with various species of microorganisms can decrease the cytotoxicity of herbal extracts or generate a wide spectrum of antibacterial activities [9, 10]. For example, some probiotics have the potential to produce new antioxidative ingredients or reduce the cytotoxicity of herb extracts by fermentation [3, 11].

*Magnolia officinalis* Rehd. et Wils. is a member of the Magnoliaceae family. The Chinese name of the bark of* M. officinalis* is called Houpo. Pharmacological studies have indicated that* M. officinalis* has antioxidative, antispasmodic, anticancer, and antidiabetic activities [12, 13]. In traditional Chinese medicine, the roots, stems, and branches of* M. officinalis* have been used for treating cough, asthma, liver disease, and diarrhea [14]. Furthermore,* M. officinalis* has shown potential antibacterial activity against methicillin-resistant* Staphylococcus aureus* (MRSA), which is a common cause of multidrug-resistant infections with considerably high mortality rates [15]. Ding et al. (2011) reported that* M. officinalis* extracted with 95% ethanol exhibited melanogenesis inhibition in murine melanoma cells [16]. Fermented* M. officinalis *extracts contain the release of functional ingredients from the unfermented extracts and enhance antioxidant activity [10]. However, fermentation is not a panacea; the choice of an appropriate species is also necessary to obtain high physiological activity.* A. oryzae-*fermented* M. officinalis* extract was reported to exhibit negligible antioxidant activity [10]. Nevertheless, few reports have demonstrated the physiological activities of fermented* M. officinalis* extracts in terms of tyrosinase inhibitory activity, antioxidant activity, antimicrobial activity, antiaging activity, and melanogenesis inhibition in “human” melanoma cells.

Our previous studies have shown that fermentation with probiotic bacteria significantly improves the tyrosinase inhibitory activity and antioxidant activity of some herb extracts, demonstrating the ability to produce various bioactive compounds through different metabolic pathways by using fermentation [3, 17]. In this study,* M. officinalis* was separately extracted with water, methanol, and ethanol. Subsequently, the* M. officinalis* methanolic extract was fermented by* Aspergillus niger.* The tyrosinase inhibitory activity, antioxidant activity, and antimicrobial activity of the unfermented and* A. niger*-fermented extracts were exhaustively evaluated. The reducing power, Fe(II) chelating (FIC) ability, phenolic composition, and contents of these extracts were also analyzed. Additionally, the effects of the* M. officinalis* extracts on cytotoxicity, melanin production, and skin-aging-related enzymes in human skin cells were examined. According to our review of the literature, this study is the first to demonstrate significant antiaging activity of fermented MOB extracts and to evaluate the inhibition of melanin synthesis in “human” HEMn by* A. niger*-fermented MOB extracts.

## 2. Material and Methods

### 2.1. Chinese Herb, Microorganisms, Test Cell Lines, and Tyrosinase

*Magnolia officinalis *Rehd. et Wils. was provided from a vendor on Dihua Street, Taipei City, Taiwan, and identified by Professor Bau-Yuan Hu. A voucher specimen (20151030) was deposited in the herbarium of China University of Science and Technology, Taiwan.* Aspergillus niger* (ATCC 42418),* Escherichia coli* (ATCC 8739),* Staphylococcus aureus* (ATCC 6538),* Bacillus subtilis* (ATCC 39093), MRSA (ATCC 33591),* Propionibacterium acnes* (ATCC 6919),* Staphylococcus epidermidis* (ATCC 14990),* Epidermophyton floccosum* (ATCC 18397), and cultures of human epidermal melanocytes (HEMn) from neonatal foreskin propagated in medium 254 (Cascade Biologics, Inc., Portland, USA) containing human melanocyte growth supplement (Cascade Biologics, Inc., Portland, USA) and the normal human skin fibroblast cell line CCD-966SK (ATCC CRL-1881) were purchased from the Bioresource Collection and Research Center (Hsinchu, Taiwan). Mushroom tyrosinase was purchased from Sigma Chemical Co. (St. Louis, USA) [3]. All chemicals used in the experiment were analytical grade (purity >99%) and obtained from Sigma-Aldrich (St. Louis, USA).

### 2.2. Extraction and Fermentation of M. officinalis

300 g of 0.3 mm dried bark powder was extracted using three solvents: distilled water (w/v=1/10), 95% ethanol (w/v=1/3), and methanol (w/v=1/3). First, the solvent containing* M. officinalis* powder was sonicated at 40°C for 2 h. Then, the extracts were filtered and concentrated in a rotary vacuum evaporator at 50°C. The residue was freeze-dried and then refrigerated until further use [3].

*A. niger* was cultured in potato dextrose broth (PDB) at 24°C for 5 d. For fermentation, sterile PDB (50 mL) containing* M. officinalis* extracts (0.5 g) was inoculated with 1 mL of* A. niger *spore suspension (1×10^7^ spores/mL). These mixtures were incubated at 24°C in an orbital shaking incubator for 9 d. The optimal fermentation periods for* M. officinalis* extracts were evaluated by their antityrosinase and antioxidant activities.

### 2.3. Analysis of Biofunctional Activity of M. officinalis Extracts

After fermentation, the solution was centrifuged at 8,000 ×g for 25 min, and the supernatant was collected, filtered, and concentrated in the rotary vacuum evaporator at 50°C. The residues were freeze-dried and stored under refrigeration [3].

### 2.4. Analysis of Antityrosinase Activity

To determine the antityrosinase activities of the* M. officinalis* extracts, the method described by Zheng et al. (2012) was used [18]. First, the extracts were dissolved in dimethyl sulfoxide (DMSO) and diluted to different concentrations. Subsequently, 30 *μ*L of the resulting mixture was mixed with 970 *μ*L sodium phosphate buffer (0.05 mM), and 1 mL of 100 mg/L l-tyrosine and 1 mL of mushroom tyrosinase solution (350 units/mL) were next added. This reaction solution was homogeneously mixed, and the initial absorbance was measured at 490 nm using a UV–vis spectrophotometer (Shimizu, Japan). The final absorbance of the solution was measured after 20 min of incubation. The concentration at which half the original tyrosinase activity was inhibited (IC_50_) was calculated for fermented and unfermented* M. officinalis* extracts. The antityrosinase activity of the* M. officinalis* extracts is expressed as a percentage of tyrosinase inhibition as follows:

(1) Tyrosinase inhibition %=A−B−C−DA−B×100,

where A is the absorbance at 490 nm without the extracts (control), B is the absorbance at 490 nm without the extracts and enzyme (blank), C is the absorbance at 490 nm with the extracts and enzyme (experimental group), and D is the absorbance at 490 nm without the enzyme (blank of C).

### 2.5. Analysis of Antioxidant Activity

To determine the antioxidant activities of the* M. officinalis* extracts, the method described by Chen et al. (2012) was used [19]. A stock solution of 2,2-diphenyl-1-picrylhydrazyl (DPPH) at 100 *μ*M was prepared in pure ethanol (97%).* M. officinalis *extracts at different concentrations (1 mL) were individually added to ethanol (1 mL) and the DPPH solution (500 *μ*L). The absorbance of this mixture was read at 517 nm versus a blank without the* M. officinalis* extracts after 1 h incubation at 25°C in the dark. The scavenging activities of the DPPH radical or antioxidant activities of the fermented and unfermented* M. officinalis *extracts are calculated as follows:

(2) $$\begin{array}{c} \text{DPPH scavenging activity }\left(\;\%\;\right)=\left(\;\frac{A_{0}-A}{A_{0}}\;\right)\times 100, \end{array}$$

where A_0_ is the absorbance of the blank (without extract) and A is the absorbance of the test sample. The IC_50_ values of the DPPH radical by the extracts were evaluated at 50% scavenging activity.

### 2.6. Analysis of Reducing Power

To determine the ferric reducing power of the* M. officinalis* extracts, the method described by Fejes et al. (2000) was applied [20]. Various concentrations of the extracts (1 mL) were mixed with 2.5 mL of 0.2 M phosphate buffer and 2.5 mL of 1% potassium ferricyanide. The mixture was incubated at 50°C, and 2.5 mL of 10% trichloroacetic acid was then added to the solution. The reaction solution was next centrifuged at 4,000 ×g for 20 min to collect its supernatant. Subsequently, 2.5 mL of the collected solution was mixed with 0.5 mL of ferric chloride (0.1%) and 2.5 mL of deionized water. The absorbance of the reaction solution was measured at 700 nm after a 10 min reaction. The concentrations of* M. officinalis* extracts providing 0.5 of absorbance (i.e., IC_50_) were calculated from the graph of absorbance at 700 nm versus the concentrations of the* M. officinalis *extracts in the solution.

### 2.7. Analysis of Ferrous Ion-Chelating (FIC) Ability

To determine the FIC ability of the* M. officinalis* extracts, the method described by Chan et al. (2010) was applied [21]. 2 mL of* M. officinalis *extracts at different concentrations was mixed with 0.1 mL of FeSO_4_ (2 mM) and 0.2 mL of ferrozine solution (5 mM). The absorbance of the solution was measured at 562 nm after 10 min reaction at room temperature. FeCl_2_ and ferrozine were used as a control. The FIC ability of extracts to chelate ferrous ions was calculated as follows:

(3) $$\begin{array}{c} \text{Chelating ability }\left(\;\%\;\right)=\left(\;\frac{1-A_{sample}}{A_{control}}\;\right)\times 100, \end{array}$$

The FIC assay results are expressed as chelating IC_50_ values (in mg/L).

### 2.8. Analysis of Phenolic Compounds in M. officinalis Extracts

Total phenolic content in the unfermented and fermented* M. officinalis* extracts was estimated as gallic acid equivalents according to the method of Cai et al. (2004), with minor modifications [22].* M. officinalis *extracts were mixed with 1 mL of a Folin–Ciocalteu phenol reagent and 1 mL of a Na_2_CO_3_ solution, and then the mixture was shaken for 10 min. Absorbance was measured at 725 nm after 60 min incubation. The regression equation between absorbance and concentration of gallic acid was calculated as* y*=0.0426*x*+0.0812 (r^2^=0.9952). The total phenolic content was expressed as the gallic acid equivalent (mg-GAE/g-dried extract).

To determine the phenolic compositions of the unfermented and fermented* M. officinalis* extracts, a high-performance liquid chromatography (HPLC) method modified from Cai et al. (2004) was applied [22]. These extracts were first dissolved in methanol, transferred to vials, and filtered through a 0.45-*μ*m filter before injection into a HPLC system (Hitachi, Japan). The operational column, flow rate, injection volume, and column temperature were as follows: 4.6 mm × 250 mm Econosil column (5 *μ*m), 1.0 mL/min, 25 *μ*L, and 20°C, respectively. The separation was performed with gradient elution (solution A, 50 mM sodium phosphate in 10% methanol, pH 3; and solution B, 70% methanol) as follows: 0 min, 100% A; 10 min, 70% A; 40 min, 60 %; 60 min, 50% A; 70 min, 40% A; and 90 min, 0% A. The detection wavelengths were adjusted from 230 to 420 nm to analyze different compounds. Individual phenolic compounds were collected and identified by comparing their retention times against those of the standard samples.

### 2.9. Effect of M. officinalis Extracts on the 3-(4,5-dimethylthiazol-2-yl)-2,5-diphenyltetrazolium Bromide Assay and Cellular Melanin Content in HEMn

The cytotoxicity levels of the* A. niger*-fermented* M. officinalis* extracts on HEMn and CCD-966SK cells were assessed through the 3-(4,5-dimethylthiazol-2-yl)-2,5-diphenyltetrazolium bromide (MTT) method. The MTT assay was performed to examine the viability of cells, and the examination method was modified from that described by Liao et al. (2012) [23]. After 24 h of incubation, the cells (3 × 10^6^ cells/wel1) were washed in fresh medium and treated with the culture medium or different concentrations (0–500 mg/L) of fermented extracts for 72 h. Moreover, after 24, 48, and 72 h of treatment, MTT was added at a final concentration of 500 mg/L at 37°C. After 2 h of MTT treatment, media were removed and the precipitate in each dish was dissolved in 100 *μ*L of DMSO. The dishes were gently shaken for 20 min, after which the absorbance of the supernatant was measured at 595 nm using a microplate reader (Plate Chameleon V, Hidex, Finland). The amount of viable cells after each treatment was expressed as the percentage of the control.

The melanin content in HEMn was measured according to the method of Liao et al. (2012) [23]. Briefly, HEMn (2 × 10^6^ cells/well) were incubated in six-well culture plates and treated with* A. niger*-fermented* M. officinalis* extracts at various concentrations (0–200 mg/L) for 24 h. Cell pellets were lysed with 1 N NaOH containing 10% DMSO and heated at 80°C for 1 h; suspensions were clarified by centrifugation for 10 min at 10,000 ×g. Relative melanin content was measured at 450 nm using an ELISA plate reader. The melanin content was measured by comparison with a synthetic melanin standard.

### 2.10. Effect of M. officinalis Extracts on Minimum Inhibitory Concentration (MIC)

MIC was determined through a microdilution method using serially diluted herb extract according to the method described by Rahman et al. (2013) [24]. The MICs of six strains of bacteria (*E. coli, S. aureus, B. subtilis, *MRSA,* P. acnes*, and* S. epidermidis*) and one strain of fungus* E. floccosum* were determined through the dilution of the* M. officinalis* extracts at different concentrations (10–20,000 mg/L). Equal volumes of each extract and specific broth were mixed in a test tube. Specifically, 0.1 mL of standardized inoculum (10^7^ cfu/mL) was added to each tube. Two control tubes were maintained for each test. These were antibiotic control (tube containing extract and the growth medium without inoculum) and microbial control (the tube containing the growth medium, physiological saline and the inoculum). The lowest concentration of the extract at which no visible bacterial growth was found compared with the control tubes was considered the MIC.

### 2.11. Effect of M. officinalis Extracts on Skin Aging Enzymes

*Analysis of Collagenase Activity and Elastase Activity. *Collagenase activity was measured using a modified fluorogenic DQ™-gelatin assay, as described by Vandooren et al. (2011) [25]. Briefly, various concentrations of* M. officinalis* extracts were added to 96-well plates. Subsequently, 1 U/mL of collagenase was added to each well (100 *μ*L/well). DQ gelatin (15 *μ*g/mL) was then added and the mixtures reacted for 15 min. The rate of proteolysis was determined by measuring the absorbance at an excitation wavelength of 485 nm and an emission wavelength of 528 nm.

The elastase activity assay was modified from Karim et al. (2014) [26]; 20 *μ*L of extracts was diluted with 50 *μ*L of buffer solution containing 100 mM HEPES, 500 mM NaCl, and 0.05% Tween 20 in DMSO in a 96-well plate. Elastatinal (100 *μ*M) was used as the control inhibitor. The neutrophil elastase enzyme was added to the diluted* M. officinalis* extracts and reacted for 10 min at 37°C. Subsequently, 5 *μ*L of substrate (MeOSuc-Ala-Ala-Pro-Val-pNA) was added to each well, and absorbance was monitored at 405 nm.

*Analysis of MMP-1 Activity and MMP-2 Activity. *Quantitative enzyme-linked immunosorbent assay (ELISA) was used to determine extract-induced MMP-1 expression in the CCD-966SK cells using ELISA kits (R&D, USA), as described by Tsai et al. (2014) [27]. Test samples of 100 *μ*L were added to 96-well plates for 24 h at 4°C. The wells were blocked with bovine serum albumin and incubated with the respective antibodies for 1 h at 26°C. The plates were then washed with wash buffer, incubated with secondary antibodies linked to peroxidase for 1 h at 26°C, washed again, and incubated with peroxidase substrate until the development of color, which was measured spectrophotometrically at 450 nm.

MMP-2 activity was assayed using gelatin zymography [28]. CCD-966SK cells were cultured in DMEM serum-free medium for 24 h. Subsequently, the culture supernatant was collected and applied to 10% polyacrylamide gels containing 0.1% w/v of gelatin. The gels were washed twice with 2.5% v/v of Triton X-100 for 30 min at 26°C to remove sodium dodecyl sulfate. Each gel was cut into slices, and the slices were placed in different tanks and incubated with activation buffer (50 mM Tris-HCl, 200 mM NaCl, 10 mM CaCl_2_, pH 7.4) containing various concentrations of* M. officinalis* extracts at 37°C for 24 h. The gels were then washed and stained with Coomassie Brilliant Blue R (0.1% w/v) and then destained in 30% methanol and 10% acetic acid. MMP-2 activity appeared as a clear band against a blue background. Digestion bands were quantitated by the Image J program.

### 2.12. Statistical Analysis

Experimental results in this study were reported as means ± standard deviation of three replicates. Statistical analysis was performed with one-way ANOVA followed by Duncan's multiple range test. The level of statistical significance was set at* P *< 0.05 or < 0.01 using SPSS version 20.0 (SPSS Inc. Chicago, IL, USA). The IC_50_ values were calculated by using Origin software.

## 3. Results and Discussion

### 3.1. Optimal Solvent Selection, Tyrosinase Activity Inhibition, and Antioxidant Activity

**Figure 1:**
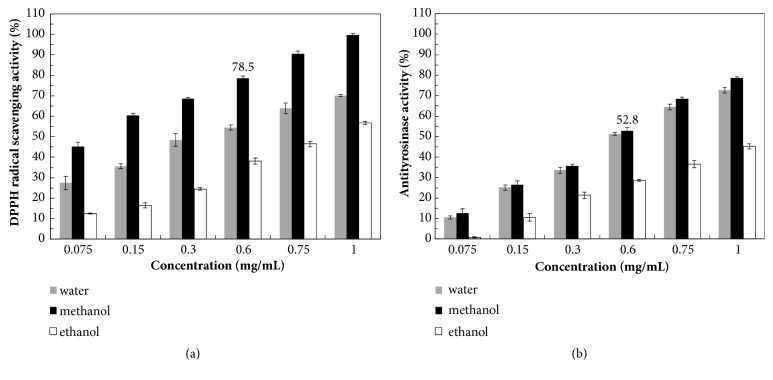
(a) DPPH radical scavenging activity of* Magnolia officinalis* extracts by different solvents. (b) Antityrosinase activity of* Magnolia officinalis* extracts by different solvents. Data are expressed as the means ± standard deviations of 3 independent experiments.

Figure 1 presents the effects of different solvent extracts on DPPH radical scavenging activity and antityrosinase activity. Before fermentation by* A. niger*, DPPH radical scavenging activity and antityrosinase activity increased with the concentration of the* M. officinalis *extracts. Methanol was the optimal extraction solvent for the bioactive compounds of* M. officinalis*; the methanol extracts showed the highest DPPH radical scavenging activity and antityrosinase activity among the three different solvents (water, methanol, and ethanol). This indicates that the selection of an appropriate solvent is critical to achieving optimal extraction yield and desired physiological characteristics of the extract [17, 29]. To further enhance the biofunctional activities of the methanol* M. officinalis *extracts, the subsequent experiments were conducted using methanol extracts to ferment.

**Figure 2:**
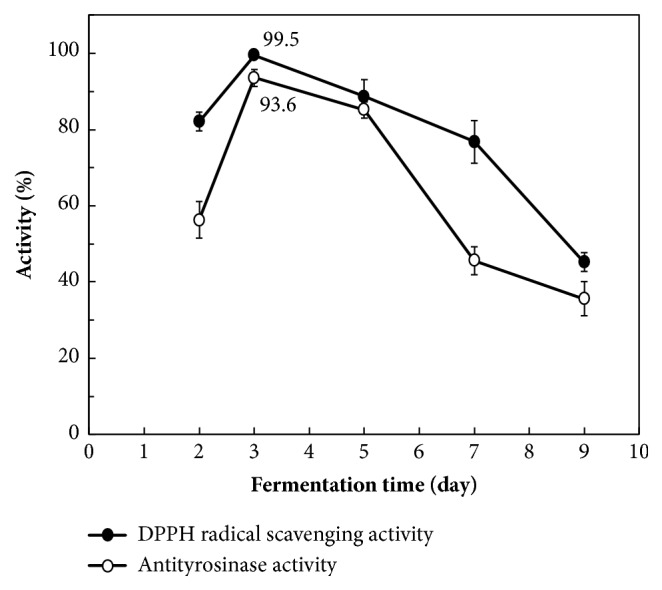
DPPH radical scavenging activity and antityrosinase activity of* Magnolia officinalis* extracts fermented by* Aspergillus niger* for different days. Data are expressed as the means ± standard deviations of 3 independent experiments.

After fermentation by* A. niger*, the fermented extracts (0.6 mg/mL) had the highest DPPH radical scavenging activity and antityrosinase activity at day 3 (Figure 2). The highest DPPH radical scavenging activity increased from 78.5%  ± 1.2% (before fermentation) to 99.5%  ± 0.2% (after fermentation), and the highest antityrosinase activity increased from 52.8%  ± 1.5% (before fermentation) to 93.6%  ± 1.8% (after fermentation). Therefore, in the subsequent experiments, a 3-day fermentation period was used to evaluate the physiological characteristics of the extracts. A study on fermented* Magnolia denudata*, which belongs to the same genus as* M. officinalis*, also revealed an optimal fermentation time of 3 days [30].

Table 1 lists the extraction yields, tyrosinase inhibitory activity, total phenolic content, DPPH radical scavenging activity, reducing power, and FIC ability of the* M. officinalis* extracts obtained using the various solvents. The extraction yield for the solvents increased in the following order: methanol (41.52%  ± 2.61%) > ethanol (33.62%  ± 1.38%) > water (28.76%  ± 1.82%). Additionally, the* A. niger*-fermented extracts exhibited significantly higher total phenolic content and antioxidant properties (DPPH scavenging activity, reducing power, and FIC ability) compared with the unfermented extracts. The* A. niger*-fermented extracts also showed 2.15- and 4.29-fold greater total phenolic content in an ethyl acetate extract of* M. liliiflora *[31] and in a* Pediococcus acidilactici*-fermented* M. denudata* ethanol extract [30], respectively. The increases in the total phenolic content of the* M. officinalis* extracts following fermentation are consistent with the findings for litchi pericarp polysaccharide [32]. Zengin et al. (2015) also reported that the tyrosinase inhibitory activity and antioxidant activity of plant extracts were strongly positively correlated with their total phenolic content [33].

The tyrosinase inhibitory activity of the* A. niger*-fermented extracts was higher than that of the unfermented extracts and the positive control, arbutin (IC_50_, 0.056 ± 0.012 mg/mL), but lower than that of kojic acid (IC_50_, 0.018 ± 0.005 mg/mL). Hsieh et al. (2015) also observed a similar tendency for many traditional Chinese medicine products [34]. The DPPH radical scavenging activity and reducing power of the* A. niger*-fermented extracts were significantly superior to those of the control BHT (IC_50_, 1.13 ± 0.31 mg/mL) and BHT (IC_50_, 3.06 ± 0.51 mg/mL), respectively. The FIC activity of the* A. niger*-fermented extracts was inferior to that of the control EDTA (IC_50_, 0.01 ± 0.006 mg/mL).

### 3.2. Phenolic Content and Phenolic Compound Identification

The results of our previous studies have suggested that high total phenolic content of fermented herb extracts resulted in high antityrosinase activity and DPPH scavenging activity. To enhance our understanding of the phenolic composition of the* M. officinalis* extracts, we analyzed the fermented and unfermented extracts using HPLC to identify the phenolic compounds.  Table 2 lists the 14 types of detectable phenolic compounds in the fermented and unfermented extracts and their contents. The results revealed that the composition of the detectable phenolic compounds increased from 11 to 14 types of compounds through fermentation, and honokiol (264 ± 1.53 to 317 ± 1.18 *μ*g/g-extract) and magnolol (187 ± 0.88 to 312 ± 1.16 *μ*g/g-extract) were predominant among the fermented and unfermented extracts. Honokiol and magnolol are two isomers from lignans isolated from* M. officinalis, *which show some pharmacological activities such as antioxidant, antitumor, and antimicrobial activities [35]. These results suggest that certain phenolic compounds are generated and some phenolic compounds are transformed after fermentation. Regarding the pharmaceutical effects, quercetin can inhibit tyrosinase activity and bacterial activity [26, 36]. Catechin, ferulic acid, and chlorogenic acid exhibit antiaging and antibacterial activities. Compared with the unfermented extracts, the physiological activities of the* A. niger*-fermented extracts were significantly improved because the concentrations of honokiol, magnolol, quercetin, and chlorogenic acid increased 1.2–2.8-fold and two new products, namely, catechin and ferulic acid, were generated.

### 3.3. Assessment of Human Skin Fibroblast Cell Viability

**Figure 3:**
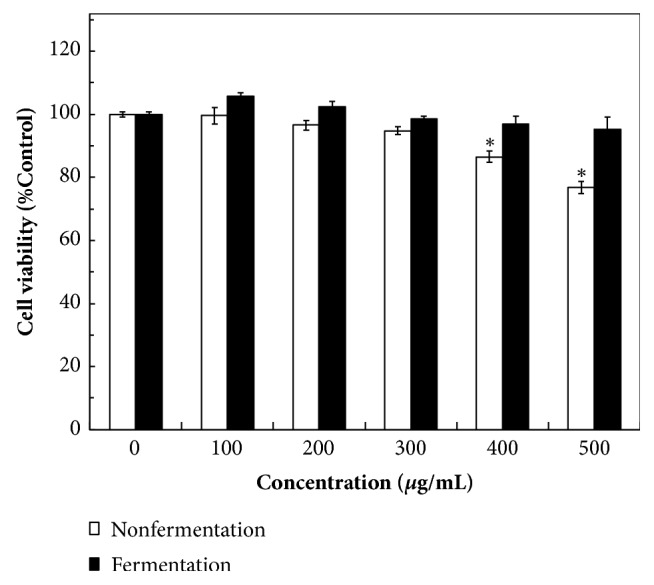
Cell viability analysis of the CCD-966SK cells treated by unfermented and fermented* Magnolia officinalis* extracts with various concentrations (0–500 *μ*g/mL) for 72 h. Data are expressed as the means ± standard deviations of 3 independent experiments (*∗P* < 0.01 vesus blank control).

To evaluate user safety, the cytotoxic effects of unfermented and fermented* M. officinalis* extracts on CCD-966SK cells were assessed using the MTT method. The growth of CCD-966SK cells was measured after treatment for 24, 48, and 72 h, and only 72 h treatment results are shown. At lower concentrations (0–300 *μ*g/mL), the measured cell viability exceeded 94.5% and cytotoxicity was nonsignificant compared with the control (Figure 3). When the concentrations of unfermented extracts were at 400–500 *μ*g/mL, cell viability was significantly inhibited (86.5%  ± 1.8% to 76.8%  ± 2.0%) compared with the control (*P *< 0.01). The* A. niger*-fermented extracts had a small effect on cell viability (95.2±3.8%) even when the extract concentration increased to 500 *μ*g/mL (Figure 3). According to the results in Table 1, the IC_50_ values for tyrosinase inhibition, DPPH removal, reducing power, and FIC activity of the* A. niger*-fermented extracts were 0.03 ± 0.008, 0.012 ± 0.005, 0.23 ± 0.15, and 0.16 ± 0.01 mg/mL, respectively. At these IC_50_ values, the* A. niger*-fermented extracts could not inhibit the viability of CCD-966SK cells even if a concentration to achieve 100% physiological activity was used. Thus, the* A. niger*-fermented MOB extracts are safe for possible applications in the health food or cosmetics industries.

### 3.4. Assessment of Cell Viability and Melanin Content in HEMn

**Figure 4:**
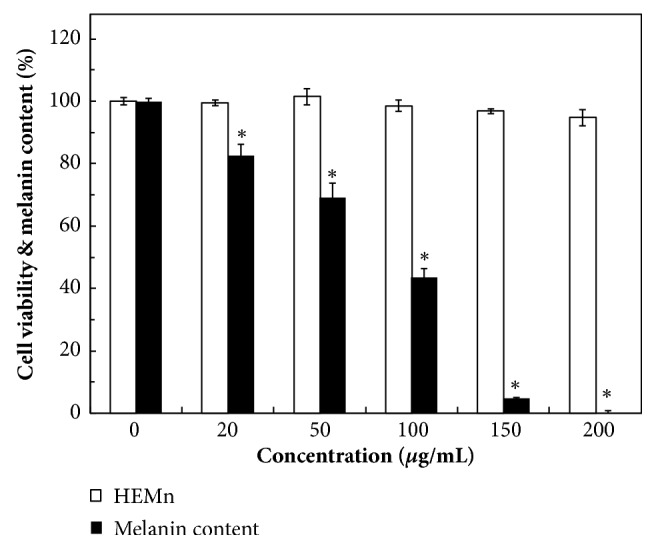
Cell viability and melanin content analysis of the HEMn cell treated by* A. niger*-fermented MOB extracts with various concentrations. Data are expressed as the means ± standard deviations of 3 independent experiments (*∗P* < 0.01 versus blank control).

The effects of various concentrations of the* A. niger*-fermented extracts on cell viability and melanin content were simultaneously evaluated in HEMn. At a concentration of 200 *μ*g/mL, the fermented extracts did not substantially harm the HEMn cell viability (94.8%  ± 2.6%) and cytotoxicity was nonsignificant compared with a control (*P *< 0.01) (Figure 4). The inhibition of melanin production in HEMn was dose dependent. The fermented extracts inhibited 30.8% of melanin formation at 50 *μ*g/mL. Limited melanin content (0.2%) was detected when the concentration of the extracts reached 200 *μ*g/mL (Figure 4). This suggests that the decrease in melanin production may be attributed to tyrosinase or other melanogenic enzymes being inhibited, not the melanocytes being killed. In the* in vitro* experiments and the results of which are summarized in Table 1, the IC_50_ of the* A. niger*-fermented extracts was 30 ± 8 *μ*g/mL for mushroom antityrosinase activity suggesting that antityrosinase activity could be theoretically achieved at 60 *μ*g/mL. Moreover, in the* in vivo* experiments using HEMn, 150 *μ*g/mL of fermented extract was required to inhibit 95.2%  ± 0.3% tyrosinase activity or melanin production. The cellular antityrosinase activity of the fermented extract was significantly lower than its mushroom antityrosinase activity. Thus, the high mushroom antityrosinase activity was not replicated in melanocytes. Huang et al. (2012) reported that a* M. grandiflora* flower extract suppressed tyrosinase activity in murine melanoma B16F10 cells (IC_50_, 13.6%) [37]. However, because humans are physiologically different from mushrooms and mice, cellular tyrosinase inhibition assays should be evaluated in human melanocyte cells [38].

### 3.5. Antimicrobial Activity

The* A. niger*-fermented MOB extracts were tested for their antimicrobial activity against some bacteria and fungus to evaluate their possible clinical application. Previous studies have reported that many phenolic compounds in herbs play a major role in antimicrobial effects [39]. The antibacterial activity of the* A. niger*-fermented extracts was significantly increased 8–20-fold compared with that of the unfermented extracts. The* A. niger*-fermented extracts at 500 *μ*g/mL were not cytotoxic against CCD-966SK cells (Figure 3). The MIC values of three food-borne bacterial pathogens* E. coli, S. aureus, *and* B. subtilis* [40] were less than or equal to 500 *μ*g/mL (Table 3); thus, the fermented extracts could be safely used as natural food or cosmetic preservatives. MRSA is the cause of nosocomial infections generally resistant to multiple antimicrobial drugs [41]. The MIC of MRSA for the* A. niger*-fermented extracts was 850 ± 122 *μ*g/mL, which is strongly effective compared with that of the unfermented* M. officinalis* extract (35,000 *μ*g/mL) [15]. Because the MIC of MRSA was >500 *μ*g/mL, the fermented extracts have the potential for application as an antibacterial ingredient. The MIC values of the human skin pathogen* P. acnes* [42] and normal human skin colonizer* S. epidermidis* [43] were <500 *μ*g/mL; therefore, the fermented extracts could be safely used as a cosmetic ingredient to prevent acne and psoriasis. The MIC (10,500 ± 1,225 *μ*g/mL) of the fungus* E. floccosum* was much higher than 500 *μ*g/mL; hence, the possible application in treating skin disorders would be restricted. Guerra-Boone et al. (2013) found that* M. grandiflora* oil displayed antifungal activity against five dermatophyte strains but low antioxidant activity [44]. These effective antibacterial activities against various bacterial strains including MRSA were due to the enhancement of concentrations of antimicrobial compounds in the fermented extract (e.g., chlorogenic acid, honokiol, magnolol, and quercetin) and production of new compounds with antimicrobial activity (e.g., catechin and ferulic acid) by* A. niger* fermentation.

### 3.6. Effect of M. officinalis Extracts on Collagenase, Elastase, MMP-1, and MMP-2 Activities

Collagenase is the enzyme that digests the triple-helix structure of collagen, which is the major foundation of the ECM in the dermis layer of the skin [45]. Therefore, the inhibition of collagenase activity could protect against collagen breakdown. Elastase is the proteinase enzyme capable of degrading elastin; hence, elastase activity inhibition could be used as a method to protect against skin aging [46]. The collagenase and elastase activities were strongly inhibited 5.65–6.88-fold by the* A. niger*-fermented extracts compared the unfermented extracts (Table 4). Furthermore, catechin found in the* A. niger*-fermented extracts (Table 2) was reported to have an inhibitory effect on elastase activity [26]. These results thus suggest that the fermented extracts could be applied to the skin surface to reduce wrinkle formation.

MMP-1 and MMP-2 are enzymes involved in the breakdown of the ECM and play major roles in affecting normal homeostasis, aging of the skin, and wound healing [4]. MMP-1 and MMP-2 secreted from skin fibroblast cells can digest collagen and gelatin, respectively [47]. The viability of CDD-966SK cells was significantly reduced when the concentration of the unfermented extracts was ≥400 *μ*g/mL (Figure 3). Thus, the actual IC_50_ values of MMP-1 and MMP-2 activity could not be obtained/measured, implying that the unfermented extracts would kill the cells. By contrast, the levels of MMP-1 and MMP-2 activity were significantly inhibited by the* A. niger*-fermented extracts (Table 4). Previous studies have reported that honokiol and magnolol could significantly downregulate the expression of MMP-1 and MMP-2 [48, 49]. This thus explains the significant antiwrinkle activity of the fermented extracts that had high concentrations of such phenolics (> 300 *μ*g/g-extract) (Table 1). These results strongly suggest that the* A. niger*-fermented MOB extracts can be used as a potential cosmetic ingredient to prevent skin aging and wrinkles.

## 4. Conclusions

In our study, the concentrations of original phenolics were increased and new phenolic compounds were biosynthesized after the fermentation of MOB extracts by* A. niger*, thereby significantly enhancing various physiological characteristics. In addition, the* A. niger*-fermented MOB extracts exhibited a wide spectrum antimicrobial activity, including activity against MRSA. According to our review of the literature, this study is the first to demonstrate significant antiaging activity of fermented MOB extracts by using skin-aging-related enzymes. Our results indicate that the fermented extracts at 200 *μ*g/mL could reduce 99.8% of melanin formation but had no cytotoxicity against HEMn. The fermented extracts exhibit relatively high biofunctional activity than do some well-known antioxidants and skin-whitening agents. Therefore,* A. niger*-fermented MOB extracts can be safe and efficient for use in applications for health food and skin cosmetics.

## Figures and Tables

**Table 1:** Extraction yield, tyrosinase inhibitory activity, total phenolic content, DPPH radical scavenging activity, reducing power, and Fe(II) chelating ability of *Magnolia officinalis* extracts by different solvents.

Solvent	Extraction yield (%)	Tyrosinase inhibition (IC_50_, mg/mL)		Total phenolic content (mg-GAE/g-extract)		DPPH (IC_50_, mg/mL)		Reducing power (IC_50_, mg/mL)		Fe(II) chelating ability (IC_50_, mg/mL)	
		before fermentation	after fermentation	before fermentation	after fermentation	before fermentation	after fermentation	before fermentation	after fermentation	before fermentation	after fermentation

Methanol	41.52 ± 2.61a	0.56 ± 0.04a	0.03 ± 0.008	58.6 ± 1.04a	206.5 ± 3.71	0.098 ± 0.01a	0.012 ± 0.005	1.21 ± 0.84a	0.23 ± 0.08	2.06 ± 0.31a	0.16 ± 0.01
Ethanol	33.62 ± 1.38b	1.26 ± 0.06b		26.2 ± 0.75b		0.804 ± 0.06b		3.12 ± 0.91b		4.05 ± 0.93b	
Water	28.76 ± 1.82c	0.58 ± 0.02c		32.8 ± 2.56c		0.280 ± 0.03c		2.06 ± 0.82c		2.81 ± 0.26c	

In each column different letters (a–c) mean significant differences *P* < 0.05. The *Aspergillus niger* fermentation period was 72 h.

**Table 2:** Phenolic composition and content (*μ*g/g-extract) in *Magnolia officinalis* extracts or fermented extracts. These *M. officinalis* extracts were extracted using methanol.

	Unfermented extract	Fermented extract*∗*
Apigenin	94 ± 1.03	130 ± 0.77
Caffeic acid	108 ± 1.02	148 ± 1.74
Chlorogenic acid	24 ± 0.19	67 ± 0.22
Catechin	nd	45 ± 0.13
Ferulic acid	nd	36 ± 0.21
Luteolin	57 ± 0.33	66 ± 0.27
Magnolol	187 ± 0.88	312 ± 1.16
Honokiol	264 ± 1.53	317 ± 1.18
Eucalyptol	135 ± 1.15	65 ± 0.35
Magnocurarine	87 ± 0.25	88 ± 0.32
Quercetin	56 ± 0.42	116 ± 0.23
Rhein	113 ± 0.36	215 ± 0.66
Rutin	45 ± 0.18	78 ± 0.22
Vanillic acid	nd	73 ± 0.62

nd: not detected.

*∗*The fermentation periods by *Aspergillus niger* were 72 h.

**Table 3:** Minimum inhibitory concentration (*μ*g/mL) of unfermented and fermented *Magnolia officinalis* extracts against tested bacteria and fungus strains.

	*Escherichia coli*	*Staphylococcus aureus*	*Bacillus subtilis*	Methicillin-resistant *Staphylococcus aureus* (MRSA)	*Epidermophyton floccosum*	*Propionibacterium acnes*	*Staphylococcus epidermidis*
Unfermented extract	4,000 ± 163a	6,500 ± 125a	5,000 ± 163a	16,000 ± 817a	12,000 ± 2,450a	2,000 ± 163a	5,000 ± 980a
Fermented extract	500 ± 82b	350 ± 40b	400 ± 82b	850 ± 122b	10,500 ± 1,225a	180 ± 32b	250 ± 82b

In each column different letters (a–b) mean significant differences *P* < 0.05. The *Aspergillus niger* fermentation period was 72 h.

**Table 4:** Effect (IC_50_, *μ*g/mL) of unfermented and fermented *Magnolia officinalis* extracts on skin aging enzymes. IC_50_ represents the concentration of the extracts giving 50% inhibition of the enzyme activity.

	Collagenase activity	Elastase activity	MMP-1 activity	MMP-2 activity
Unfermented extract	520 ± 48a	860 ± 32a	- - - - - *∗*	- - - - - *∗*
Fermented extract	92 ± 16b	125 ± 16b	180 ± 32	226 ± 16

In each column different letters (a–b) mean significant differences *P* < 0.05. The *Aspergillus niger* fermentation period was 72 h. MMP-1: Interstitial collagenase; MMP-2: 72 kDa-gelatinases. The IC_50_ of *Magnolia officinalis* extracts on MMP-1 activity and MMP-2 activity could not be detected.

## Data Availability

The data used to support the findings of this study are available from the corresponding author upon request.
